# Accurate Prediction of the Functional Significance of Single Nucleotide Polymorphisms and Mutations in the *ABCA1* Gene

**DOI:** 10.1371/journal.pgen.0010083

**Published:** 2005-12-30

**Authors:** Liam R Brunham, Roshni R Singaraja, Terry D Pape, Anish Kejariwal, Paul D Thomas, Michael R Hayden

**Affiliations:** 1 Centre for Molecular Medicine and Therapeutics, Department of Medical Genetics, Child and Family Research Institute, University of British Columbia, Vancouver, British Columbia; 2 Computational Biology, Applied Biosystems, Foster City, California, United States of America; University of Alabama at Birmingham, United States of America

## Abstract

The human genome contains an estimated 100,000 to 300,000 DNA variants that alter an amino acid in an encoded protein. However, our ability to predict which of these variants are functionally significant is limited. We used a bioinformatics approach to define the functional significance of genetic variation in the *ABCA1* gene, a cholesterol transporter crucial for the metabolism of high density lipoprotein cholesterol. To predict the functional consequence of each coding single nucleotide polymorphism and mutation in this gene, we calculated a substitution position-specific evolutionary conservation score for each variant, which considers site-specific variation among evolutionarily related proteins. To test the bioinformatics predictions experimentally, we evaluated the biochemical consequence of these sequence variants by examining the ability of cell lines stably transfected with the *ABCA1* alleles to elicit cholesterol efflux. Our bioinformatics approach correctly predicted the functional impact of greater than 94% of the naturally occurring variants we assessed. The bioinformatics predictions were significantly correlated with the degree of functional impairment of *ABCA1* mutations (*r* ^2^ = 0.62, *p* = 0.0008). These results have allowed us to define the impact of genetic variation on ABCA1 function and to suggest that the in silico evolutionary approach we used may be a useful tool in general for predicting the effects of DNA variation on gene function. In addition, our data suggest that considering patterns of positive selection, along with patterns of negative selection such as evolutionary conservation, may improve our ability to predict the functional effects of amino acid variation.

## Synopsis

A major goal of human genetics research is to understand how genetic variation leads to differences in the function of genes. Genome sequencing projects have generated large amounts of sequence data, yet our ability to predict which specific sequence variants will result in functional differences is currently limited. To address this problem, the authors use an evolutionary model to predict the functional significance of genetic variation in the *ABCA1* gene. To predict the functional impact of genetic variation in this gene, the authors compare the specific sites at which the variants occurred in evolutionarily related proteins and generated a likelihood score of functional impairment. These predictions were then compared to actual functional measurements of each variant. The authors show that it is possible to accurately predict which specific variants will affect ABCA1 function and to what extent. These results suggest that the evolutionary approach used may be a useful method in general for determining the functional consequence of genetic variation, which should aid in the study of how genetic variation contributes to phenotypic differences.

## Introduction

The ATP-binding cassette transporter A1 *(ABCA1)* is a cholesterol and phospholipid transporter, and mutations in *ABCA1* cause Tangier disease (TD) [1–3], a rare disorder characterized by reduced levels of plasma high density lipoprotein (HDL) cholesterol and increased risk for coronary artery disease [4]. More than 70 coding variants have been reported in the *ABCA1* gene, including 30 missense mutations, ten coding single nucleotide polymorphisms (cSNPs), and many large and small deletions and insertions [5]. Variants detected in individuals with TD have been assumed to impair the function of ABCA1. However, without functional testing of individual variants, it has not been possible to determine which of these variants directly affect ABCA1 protein function. This is a fundamental problem in human genetics, in which most DNA variants are not functionally tested and the number of individuals with any given mutation is often small, making statistical assessment difficult or impossible.

We used an evolutionary model to predict the functional consequence of genetic variation in the *ABCA1* gene and tested these predictions through in vitro assessments of protein function. We predicted the functional consequence of each variant in *ABCA1* using PANTHER [6], a collection of protein families and subfamilies that allows one to ask the question, how often does a given amino acid occur at a given position in a family of evolutionarily related proteins across different species? PANTHER uses as its dataset the natural experiment of evolution, in which over time, random mutation will test every amino acid–coding nucleotide sequence in the genome, with those variants that do not impair protein function being represented in the dataset of extant proteins. The probability that a given coding variant will cause a deleterious functional change is estimated by the substitution position-specific evolutionary conservation (subPSEC) score, derived from the probabilities of observing the variant amino acids in a PANTHER hidden Markov model (HMM).

PANTHER subPSEC scores have previously been shown to statistically distinguish Mendelian disease-associated missense mutations from random coding polymorphisms on a genomic scale [7]. Here we test the hypothesis that subPSEC scores can predict which specific variants in *ABCA1* will be functionally impaired, and to what degree.

## Results

### Prediction of Functional Effect of ABCA1 Mutations and cSNPs

**Figure 1:**
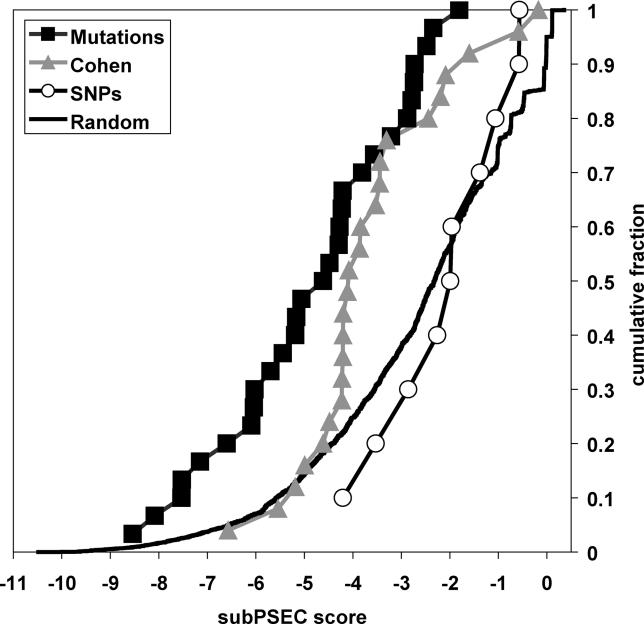
Comparison of subPSEC Scores for *ABCA1* cSNPs, Mutations, Recently Described Variants in a Cohort of Individuals with Low HDL Cholesterol from the General Population [14], and a Random Distribution of Low Frequency Alleles *ABCA1* cSNPs (open circles) have significantly greater subPSEC scores than do mutations (filled squares) (*p* < 0.0001, Mann-Whitney U test). subPSEC scores for *ABCA1* variants described in the general population (filled triangles) are significantly different from those of both *ABCA1* cSNPs and mutations (*p* < 0.01, Mann-Whitney U test), as well as from the random distribution of *ABCA1* variants (*p* < 0.001), indicating that this group of variants consists of both functional and neutral variants.

We used data from the PANTHER database to predict the functional significance of each of the 30 missense mutations and ten cSNPs reported in the *ABCA1* gene [5]. The output of PANTHER, the subPSEC score, is the negative logarithm of the probability ratio of the wild-type and mutant amino acids at a particular position. PANTHER subPSEC scores are continuous values from 0 (neutral) to about −10 (most likely to be deleterious). The subPSEC scores for *ABCA1* mutations and cSNPs are shown in Table 1. Twenty-three of the 30 *ABCA1* mutations score below −3, the previously identified cutoff point for functional significance [6], compared to two of ten cSNPs (*p* = 0.002, Fisher's exact test). The mean subPSEC score for *ABCA1* mutations is −4.82 compared to −2.03 for SNPs. Figure 1 shows the distribution of subPSEC scores for *ABCA1* mutations and cSNPs. Compared to the dataset of *ABCA1* cSNPs, *ABCA1* mutations have significantly lower subPSEC scores (*p* < 0.0001, Mann-Whitney U test). Therefore, the majority of *ABCA1* mutations are predicted to impair the function of the ABCA1 protein on the basis of the variability of the particular amino acid positions at which the variants occur in evolutionarily related proteins, compared to only a small fraction of cSNPs.

**Table 1:**
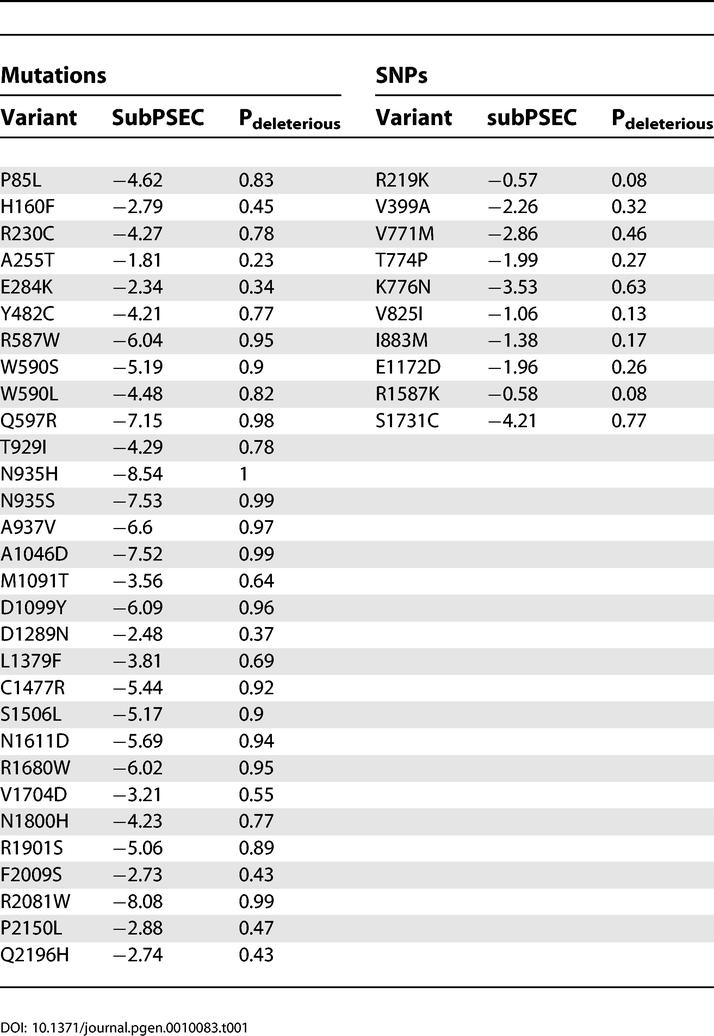
subPSEC Scores and Probability of Functional Impairment (P_deleterious_) for *ABCA1* Mutations and SNPs

### Functional Assessment of *ABCA1* Variants

In order to test experimentally the bioinformatics predictions, we established stably transfected polyclonal cell lines with which to assess cholesterol efflux as a measure of ABCA1 function. We established cell lines for 18 of the *ABCA1* alleles for which we predicted modulation of ABCA1 function using PANTHER, representing 13 mutations, four cSNPs, and wild-type *ABCA1* as a control. We choose at least one SNP and one mutation from each of the predictive categories, neutral and deleterious. In addition, we attempted to choose variants for which substantial clinical data are available in order to correlate our findings with patient phenotypes.

All *ABCA1* alleles expressed protein (R. Singaraja, H. Visscher, E. R. James, G. Chimini, and M. R. Hayden, unpublished data), with the exception of the S1731C cell line, for which we observed low levels of protein expression from two independently generated cell lines. To confirm that the S1731C allele was being expressed, we performed RT-PCR for *ABCA1* on reverse-transcribed RNA from untransfected 293 cells and cells transfected with the wild-type or S1731C *ABCA1* alleles. We found that cells transfected with the S1731C allele expressed abundant *ABCA1* mRNA, at levels comparable to that of wild-type *ABCA1* (Figure S1). The S1731C allele therefore expresses normal *ABCA1* mRNA but fails to generate significant amounts of ABCA1 protein.

We next evaluated the biochemical deficit resulting from each sequence variant by assessing apolipoprotein A-I (apoA-I)–dependent cholesterol efflux in these cell lines. Cholesterol efflux values from cell lines expressing the *ABCA1* alleles are shown in Table 2. Of the five variants we tested that were predicted to be functionally neutral (subPSEC > −3), R219K, V771M, I883M, D1289N, and P2150L, four had cholesterol efflux values that were not statistically different from wild-type *ABCA1*. This included two variants, D1289N and P2150L, that have been previously reported to be disease-causing mutations [4,8,9], as well as two cSNPs, R219K and V771M. One variant, I883M, was predicted to be functionally neutral but found to have cholesterol efflux modestly but significantly reduced (approximately 70% of wild-type *ABCA1, p* < 0.01). This SNP has been reported to be associated with decreased HDL cholesterol and increased severity of atherosclerosis in some [10,11], but not all [12], association studies, supporting the concept that this is a functional variant.

**Table 2:**
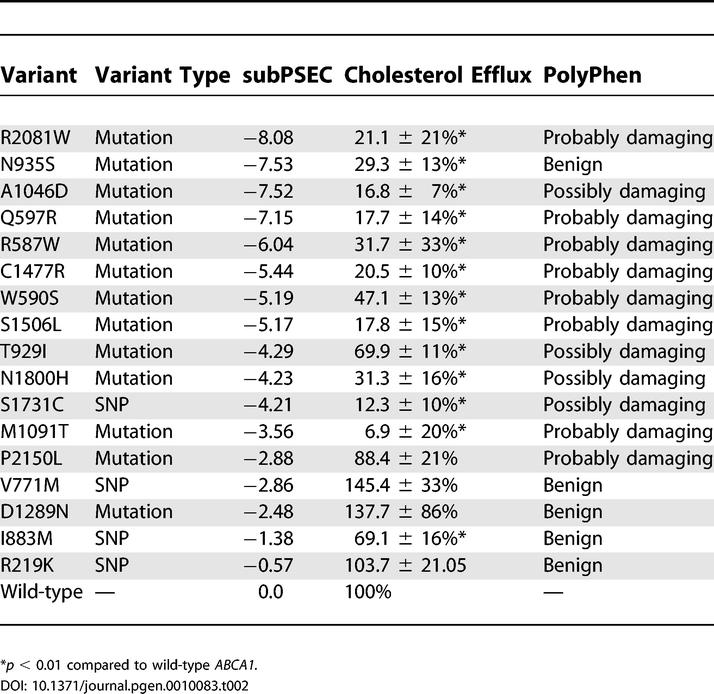
Cholesterol Efflux Values for 293 Cells Transfected with *ABCA1* Variants and subPSEC and PolyPhen Predictions of the Functional Impact of these Variants

We generated stably transfected cell lines expressing 12 different *ABCA1* variants that were predicted to impair ABCA1 function. All 12 of these variants had cholesterol efflux levels that were significantly reduced compared to wild-type *ABCA1* (Table 2), indicating that PANTHER correctly predicted the functional impact of each of these variants. The *ABCA1* cSNP, S1731C, has a subPSEC score of less than −3, predictive of a deleterious effect on ABCA1 function. Cells transfected with the S1731C allele displayed a significant reduction in cholesterol efflux, relative to wild-type *ABCA1* (*p* < 0.01), indicating that this SNP significantly impairs ABCA1 function, as predicted by PANTHER. These data indicate that S1731C may be a useful SNP to use as a functional marker in association studies.

**Figure 2:**
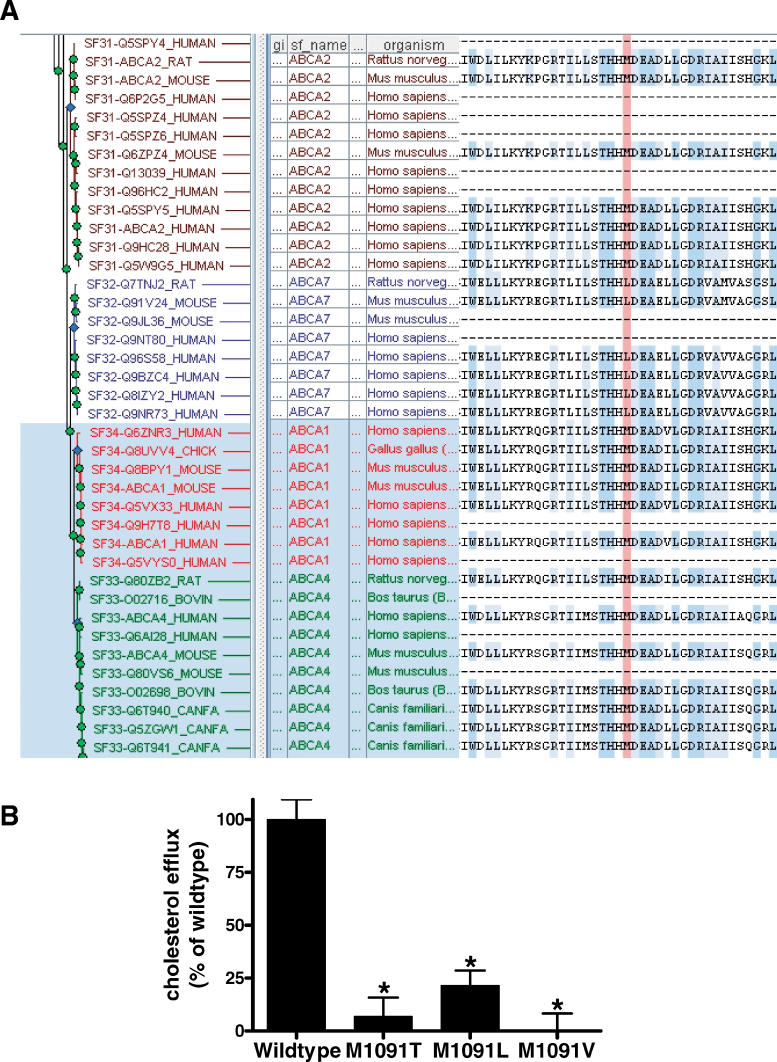
Conservation of ABCA1 Amino Acid Position 1091 in Related Proteins and Functional Effect of Mutation at This Site (A) Multiple sequence alignment adapted from the view of family PTHR19229 available on the PANTHER Web site, showing the ABCA1, ABCA2, ABCA4, and ABCA7 subfamilies. Human ABCA1 position 1091 is highlighted in red; other conserved positions are highlighted in blue. (B) Cholesterol efflux was assessed in 293 cells stably transfected with wild-type, M1091T, M1091L, or M1091V ABCA1 alleles. *p < 0.001.

Of all *ABCA1* alleles tested functionally, M1091T displays the greatest reduction in cholesterol efflux (6.9 ± 20% of wild-type *ABCA1*), consistent with previous reports that this is a severe mutation associated with a severe clinical presentation [4,13]. However, the PANTHER score for this mutation (−3.56) is only marginally predictive of a negative impact on function, because while this position is conserved in ABCA1 proteins in other species, it is less conserved among other members of the human ABCA subfamily of proteins, the orthologous position aligning a leucine in the closely paralogous ABCA7 protein (Figure 2A). To determine whether the severe phenotype conferred by the M1091T mutation is a result of the sensitivity of this site, or rather is specific to the insertion of the threonine residue, we generated and characterized cell lines transfected with plasmids bearing M1091L and M1091V alleles, both predicted to have no impact on ABCA1 function (subPSEC scores −2.65 and −2.71, respectively). Interestingly, both of these mutations dramatically impair cholesterol efflux, to a similar extent as the M1091T mutation (Figure 2B). Therefore, amino acid position 1091, occurring in the first nucleotide binding domain of ABCA1 [5], appears to be exquisitely sensitive to mutation and absolutely critical for ABCA1 function, despite its relatively modest conservation in related human ABCA proteins. This finding also supports the notion that any amino acid changes in the nucleotide binding region of ABCA1 are likely to have significant functional effects regardless of their evolutionary conservation.

**Figure 3:**
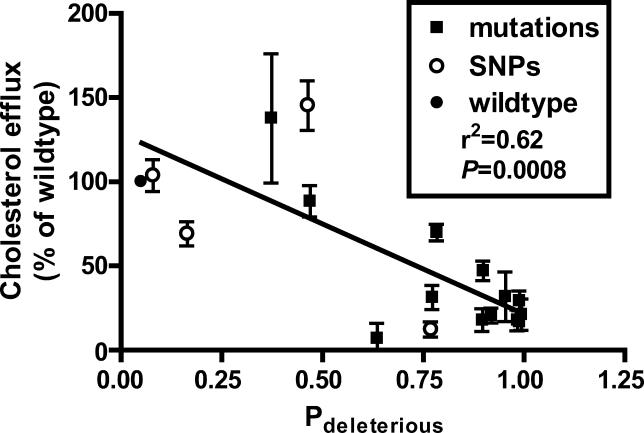
Correlation of Cholesterol Efflux Values with the Probability of a Functional Impairment (P_deleterious_) for *ABCA1* Mutations (filled squares) and SNPs (open circles) PANTHER predictions are significantly correlated with the severity of impairment of *ABCA1* mutations (*r* ^2^ = 0.62, *p* = 0.0008) and of all *ABCA1* variants (*r* ^2^ = 0.56, *p* = 0.0004). The linear regression shown is for *ABCA1* mutations.

Data from PANTHER can also be used to calculate the probability that a given variant will have a deleterious effect on protein function (P_deleterious_), such that a subPSEC score of −3 corresponds to a P_deleterious_ of 0.5 (see Materials and Methods for details). Figure 3 shows a plot of cholesterol efflux of individual *ABCA1* variants versus the probability of each variant being functionally impaired. The P_deleterious_ value is significantly correlated with cholesterol efflux for *ABCA1* mutations (*r* ^2^ = 0.62, *p* = 0.0008), indicating not only that PANTHER can discriminate between neutral and functional mutants but also that those variants with a greater P_deleterious_ tend to have more severe impairments in function. Inclusion of *ABCA1* SNPs in the linear regression also reveals a significant correlation (*r* ^2^ = .56, *p* = 0.0004), indicating that this relationship is significant across all *ABCA1* variants.

### Assessment of Rare ABCA1 Variants Identified in the General Population

Recently, Cohen et al. [14] reported that a significant proportion (~16%) of individuals with low HDL cholesterol from the general population have rare sequence variants in *ABCA1.* An important unanswered question from these data is what functional deficit results from these rare *ABCA1* alleles. We used data from PANTHER to predict the functional consequence of each of the 24 *ABCA1* alleles found in individuals with low HDL cholesterol by Cohen et al. (Table 3). This set of variants is shifted toward lower scores compared to the set of common *ABCA1* cSNPs (*p* < 0.01, Mann-Whitney U test) (Figure 1). The subPSEC scores for the Cohen et al. set of variants are also significantly greater than those observed for *ABCA1* mutations (*p* < 0.01, Mann-Whitney U test), indicating that this set of alleles is significantly different from both *ABCA1* mutations and SNPs. Fourteen of these 24 alleles (58%) have subPSEC scores less than −3, compared to 77% of *ABCA1* mutations and 20% of *ABCA1* cSNPs.

**Table 3:**
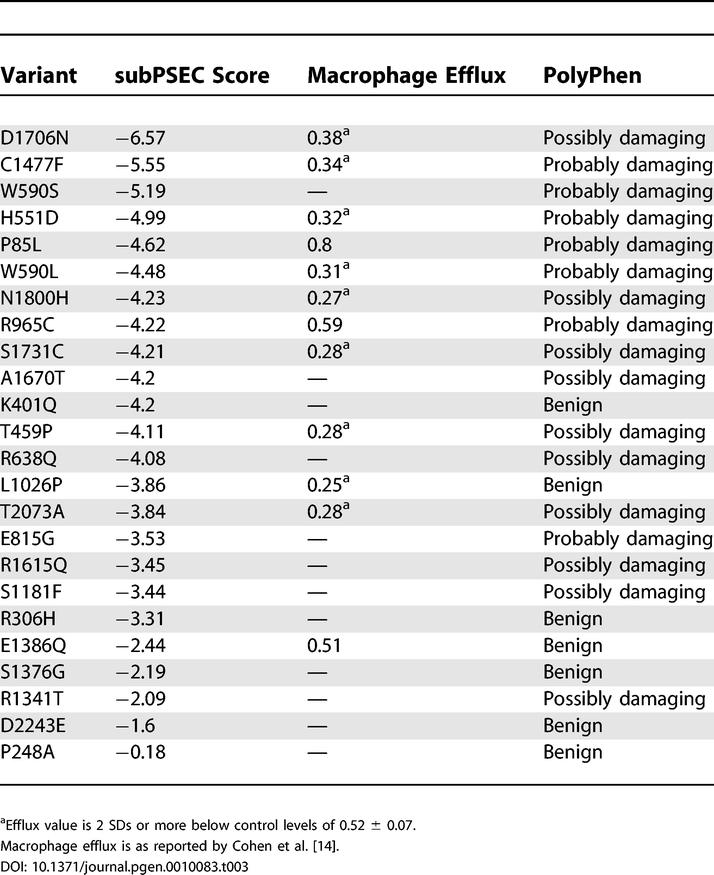
subPSEC Scores for *ABCA1* Variants Described in a Cohort of Individuals with Low HDL Cholesterol from the General Population

In order to control for the possible confounding effect of comparing subPSEC scores of variants with very different frequencies, we compared the distribution of subPSEC scores for the Cohen et al. variants to the distribution of random single-nucleotide missense changes in *ABCA1*. Recent low-frequency mutations that have not yet been subject to significant selection pressures would be expected to approximate such a random model. To estimate the resulting distribution of subPSEC scores, we calculated the scores for all single-nucleotide missense variants in *ABCA1,* weighting the contribution of each variant by transition/transversion ratios. Figure 2 shows that the distribution of subPSEC scores for Cohen et al. variants is shifted significantly toward lower scores than the random distribution (*p* < 0.001, Mann-Whitney U test). The Cohen et al. set of variants is therefore predicted to be enriched in deleterious alleles but likely to also include neutral variants.

### Comparison with PolyPhen

PolyPhen (Polymorphism Phenotyping; http://genetics.bwh.harvard.edu/pph) is a Web-based program used to predict allele function based on homology and three-dimensional structural models where available [15], and it predicts alleles as being “probably damaging,” “possibly damaging,” or “benign.” In the dataset of variants for which we assessed cholesterol efflux, the predictions made by PANTHER and PolyPhen were significantly different for two mutations: N935S and P2150L. PolyPhen predicted N935S to be benign, while PANTHER predicted it to be deleterious. Conversely, PolyPhen predicted P2150L to be probably damaging, while PANTHER predicted it to be neutral. In both of these cases, the PANTHER predictions were correct. Using the set of 12 *ABCA1* variants described by Cohen et al. [14] for which macrophage efflux is reported as a second dataset, the PANTHER prediction differs significantly from PolyPhen in one case, where PANTHER correctly predicts that L1026P will be deleterious (efflux rates ≤2 SDs below control levels), while PolyPhen predicts the substitution will be benign. Therefore, while PANTHER predictions correlate quite closely with those of PolyPhen overall, the PANTHER predictions are correct in the three instances in which they differ significantly, representing a significant difference in the ability of these two approaches to correctly identify functionally significant alleles (*p* < 0.05, Fisher's exact test).

## Discussion

By combining bioinformatics predictions with direct biochemical assessment, we have shown that it is possible to accurately predict the functional consequence of amino acid variation on protein function using an evolutionary model. We demonstrated that it is possible to differentiate cSNPs from mutations in *ABCA1* using data from PANTHER (Figure 1), indicating that *ABCA1* mutations tend to occur at much more highly conserved positions in evolutionarily related proteins compared to cSNPs. This finding within a single gene is in agreement with the genomewide finding that mutations from the Human Gene Mutation Database have lower subPSEC scores than the randomly collected SNPs from the dbSNP database [6].

We used an in vitro test of ABCA1 function in stably transfected polyclonal cell lines to determine the biochemical impact of the *ABCA1* sequence variants and to evaluate the predictions made by PANTHER. Overall, PANTHER correctly predicted the functional impact of greater than 94% (16 of 17) of the naturally occurring sequence variants that we examined. The subPSEC score cutoff of −3 suggested in the initial characterization of subPSEC scores [6] worked well for predicting functional variants in *ABCA1,* and no other cutoff would have improved the prediction accuracy. In addition, cholesterol efflux was significantly correlated with the probability of a deleterious effect for *ABCA1* mutations, as well as across all variants, indicating that PANTHER scores are a significant and reliable predictor of the degree of functional impact of ABCA1 amino acid variants.

Both the D1289N and P2150L mutations are reported as pathogenic and causative of disease in the TD patients in which they were identified [4,8,9]. However, we predicted that these variants would not impair ABCA1 function based on the variability of the sites at which they occur in evolutionarily related proteins. We were able to confirm this prediction in vitro, indicating that these mutations are benign sequence variants and are unlikely to be causal of disease. The TD patient described with the D1289N variant was also homozygous for a second mutation, R2081W [9], and our results strongly suggest that it is this second mutation, and not D1289N, that causes the phenotype observed in that patient. The molecular cause of the phenotype in patients carrying the P2150L variant remains to be determined, and it is possible that these patients harbor a second, yet unidentified coding or noncoding variant.

The amino acids at positions 1289 and 2150 are conserved among all ABCA1 orthologs we examined but not among the closely paralogous ABCA7 and ABCA4 subfamilies. Because conservation patterns in ABCA1 proteins have persisted for only a relatively short time in evolutionary history, it is difficult to determine if the conservation at a given position among ABCA1 orthologs is due to functional constraint or simply reflects random chance. Accordingly, the amino acid probability profiles for these positions are determined only from ABCA1 orthologs (see Materials and Methods), which do not contain enough sequence variability to conclusively assume functional constraint. Our efflux data showing that the D1289N and P2150L mutations are functionally neutral confirm the prediction that the conservation of these residues among ABCA1 proteins is not due to functional constraint, but rather reflects their recent common ancestry.

One *ABCA1* cSNP, S1731C, had a subPSEC score less than −3. Interestingly, this SNP has been described in a French-Canadian family that also carries the 2144X stop mutation on a separate *ABCA1* allele [10]. Individuals carrying both the 2144X mutation and S1731C had significantly lower HDL cholesterol than individuals with only the 2144X mutation, although the number of patients in each group was small [10]. Cells transfected with the S1731C allele expressed *ABCA1* mRNA at levels comparable to wild-type *ABCA1;* however, this cell line expressed low levels of ABCA1 protein and was markedly deficient in cholesterol efflux. Substitution of cysteine for serine at this residue therefore significantly impairs ABCA1 function, as predicted by PANTHER analysis, by interfering with protein expression. The mechanism by which this SNP inhibits protein expression remains to be determined but may involve expression of an unstable protein that is rapidly degraded, or interference with protein translation. The identification of S1731C as a functionally significant variant indicates that it may be a useful DNA marker to be used in association studies. These results also demonstrate that PANTHER may be a useful tool in general to identify functional SNPs that would be most useful for studying in association studies.

Recently, it was reported that rare amino acid variants in ABCA1 occur in a significant percentage of individuals from the general population with low HDL cholesterol [14]. We predicted the functional consequence of these 24 *ABCA1* sequence variants using PANTHER. The subPSEC scores of this group of variants are intermediate between and significantly different from those of both *ABCA1* mutations and SNPs. We show that the subPSEC score distribution for these variants is not due to random rare variants, which is consistent with the conclusion that these variants are not simply randomly sampled but are biased toward deleterious functional effects. Our data are therefore in agreement with Cohen et al. in that many (~58%) of these variants are predicted to impair ABCA1 function and could therefore underlie the low HDL phenotype in these patients. However, subPSEC scores for these variants are also significantly greater than the subPSEC scores for mutations involved in the Mendelian disorder TD. This suggests that a substantial proportion of these variants may not impair ABCA1 function, and therefore suggest that other genes, or other undetected *ABCA1* variants, could be responsible for the low HDL phenotype in these individuals. Consistent with this hypothesis, approximately 33% of the variants functionally tested by Cohen et al. [14] were not functionally impaired.

It is equally informative to consider the incorrect predictions made by our evolutionary conservation-based method, in order to understand the limitations of this method and to suggest how it might be improved. The incorrect predictions occurred at two positions in ABCA1, 883 and 1091. The subPSEC score for the naturally occurring M1091T mutation (−3.56) is only marginally predictive of a negative impact on function, but this variant resulted in a severe reduction in ABCA1 function, consistent with the severe phenotype observed in patients harboring this mutation [4,13]. In addition, both the M1091V and M1091L substitutions severely impaired the function of ABCA1, yet they were predicted to be functionally neutral. Among closely related ABCA1 homologs, ABCA2 and ABCA4 both share a methionine at this position, while ABCA7 substitutes a leucine. From the evolutionary tree, it is apparent that the ancestral amino acid at this position is likely to have been methionine, with ABCA7 diverging from the ancestral sequence (Figure 2A). Therefore, when calculating amino acid probabilities for position 1091, the subPSEC method includes sequences from only ABCA1 and ABCA4, which represents enough sequence variability to predict that a relatively radical mutation such as M1091T will likely be deleterious but not enough to predict that relatively conservative mutations such as M1091L or M1091V will be deleterious. Our experimental finding that M1091L severely impairs ABCA1 cholesterol efflux suggests that substitution of leucine for methionine at this position may have played an important role in the functional divergence of ABCA7 from ABCA1. Consistent with this hypothesis, ABCA1 and ABCA7 have recently been demonstrated to be functionally divergent, with ABCA7 facilitating the efflux of phospholipids but not cholesterol [16]. In addition, the two proteins mediate the formation of distinct HDL particle subpopulations [17]. Taken together, our efflux data and the functional divergence of ABCA1 and ABCA7 suggest that human ABCA1 position 1091 is a critical functional site despite the relatively modest conservation at this position.

**Figure 4:**
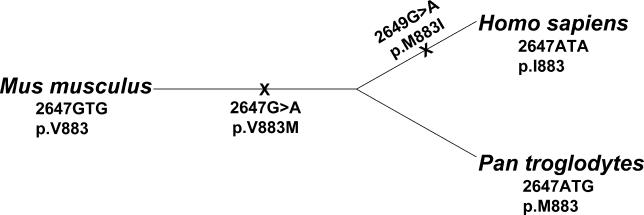
Graphic Representation of the Evolutionary Relationship between Mouse, Human, and Chimpanzee ABCA1 Proteins ABCA1 amino acid position 883 genotype is displayed under the species name. An “X” represents the likely point in evolutionary history at which the V883→M883 and M883→I883 mutation events occurred. The M883→I883 mutation likely occurred since the divergence of the last common ancestor between humans and chimpanzees, and the increased activity of the I883 allele suggests that this may have been one of the adaptive changes that occurred during the evolution of modern humans.

The I883M substitution results in a milder phenotype, with a modest but significant reduction in ABCA1-mediated cholesterol efflux. This variant is interesting, as both alleles are found in the human population and the minor allele, methionine, is likely to be the ancestral allele at this position (Figure 4). Among the human ABCA1 orthologs, murine ABCA1 aligns valine at this position and the chimpanzee sequence aligns methionine. This divergence explains why a simple conservation-based approach predicts that I883M is a neutral substitution. However, *ABCA1* has recently been shown to be among the genes most likely to have been under positive selection since the divergence of humans with chimpanzees [18]. Our experimental results showing increased efflux activity of the I883 versus M883 allele suggest that the M883→I883 mutation may have been one of the adaptive changes in ABCA1 that occurred during the evolution of modern humans. This also suggests that measures of positive selection, an approach complementary to measures of negative selection such as amino acid conservation, may be useful for identifying functionally important residues in proteins, thereby improving algorithms for predicting the functional effect of amino acid substitution.

The assumption of functional equivalence amongst homologs is fundamental to simple amino acid conservation analysis in general: the functional constraints on a position that lead to the observed conservation pattern are assumed to be constant (or approximately so) in all of the related sequences. However, there are many documented cases of missense substitutions whose phenotypic effect is highly dependent on genetic background. Among the most dramatic of these are human disease mutations that are fixed in the mouse genome with no similar phenotypic effect [19], as well as alleles that have different phenotypic effects in closely related mouse strains [20]. In addition, the degree of functional constraint on a gene is dependent on details of the population, such as effective population size, and these effects are not accounted for in simple conservation-based approaches.

It is therefore perhaps not surprising that both cases for which PANTHER analysis failed to correctly predict the functional consequence of amino acid substitution occur at positions that have apparently played a key role in the functional divergence of homologs, either paralogs (ABCA1 versus ABCA7) or even orthologs (human versus chimpanzee ABCA1). The lack of strict conservation at these positions is therefore not indicative of the lack of functional constraints; it is instead due to divergence of protein function, either to play a different role in the same organism (paralogs) or to adjust to different selective pressures in a different genetic, environmental, or population background (orthologs). Other methodologies, such as those involving measures of positive selection, will be required to recognize when an amino acid change contributes to the divergence of function.

In summary, by combining a bioinformatics approach with biochemical functional assays we have been able to define the functional significance of genetic variation in the *ABCA1* gene and to validate the use of PANTHER as a robust approach to predicting allele function. These data have contributed to our understanding of the functional effect of *ABCA1* DNA variants and, in addition, suggest that PANTHER may be a useful tool in general for predicting the functional consequence of DNA variation.

## Materials and Methods

### Datasets.

We assembled a list of 30 missense mutations and ten cSNPs reported in the *ABCA1* gene [5]. In classifying a variant as a “SNP” or “mutation,” we have relied solely on their description in the literature, with mutations being reported as variants that segregate with TD in a kindred, and SNPs being more common variants (>1% frequency) not associated with this disorder. We also examined the group of *ABCA1* variants recently reported to be associated with low concentrations of HDL cholesterol in a population based cohort [14].

### Calculation of subPSEC scores.

We used data from PANTHER [6] to predict the functional consequence of each of the *ABCA1* variants described above. The ABCA1 protein was aligned to the highest-scoring PANTHER (version 6.0, October 2005) HMMs: ABCA transporter family and ABCA1 subfamily. subPSEC score calculations were modified from previous descriptions [6,7] by using the equation subPSEC = 0.88ln(P_min_) − 0.89ln(P_max_) − 0.94ln(n_ic_), where P_min_ and P_max_ are the probabilities of the lower and higher probability amino acids that are being evaluated, respectively, and n_ic_ (number of independent counts) is the number of observations used to calculate the probabilities (B. Lazareva, A. Kejariwal, and P. D. Thomas, unpublished data). We discuss this method in more detail below. Reported subPSEC scores for nearly all missense variants are available online at the PANTHER database cSNP scoring page [21]. A small number of variants occur at positions that do not align to the PANTHER library HMM (because these positions are not present in most sequences that are members of the ABC transporter subfamily A). To calculate subPSEC scores at these positions, we built an HMM according to the method described [6], using human ABCA1 as the seed sequence. Briefly, this method forces each position in the seed sequence to be modeled as a “match state” (so that a probability vector will be calculated for every position), aligns all other sequences in PTHR19229 to the seed, and then reestimates the probability vectors using all of the aligned sequences. The alignment is not as accurate overall as the PANTHER library alignment, but we verified that it is accurate for the few positions that were not modeled by the PANTHER library alignment.

The rationale and methodology for calculating subPSEC scores have been reported previously [6,7], but we briefly describe them here, as well as describing recent improvements to the method (B. Lazareva, A. Kejariwal, and P. D. Thomas, unpublished data). The goal is to predict the functional effect of single amino acid substitutions in proteins. From genomic and cDNA sequencing, there exists a great deal of data about related protein sequences in extant organisms. To the extent that these related sequences perform the same function, they are under similar evolutionary constraints. Some of the random mutations that occur during protein sequence evolution are functionally neutral and can be fixed in extant sequences, while most mutations are selected against and will not appear in any of the extant sequences. The effect of this negative selection is apparent in the pattern of amino acids that appears in the equivalent positions in related proteins. First order HMMs have proved to be an excellent method for generating statistics on amino acid probabilities for modeling protein families [22]. In these HMMs, each equivalent position in related proteins is treated as a series of observations that were “generated” by a “hidden” model represented as a Markov chain. Each position is modeled as a vector (or “profile”) of 20 probabilities, one for each amino acid type. This profile is derived using a Bayesian method that weights prior knowledge (e.g., of physicochemical similarities between amino acids) more heavily when there are few observations. The method also weights different sequences depending on their relatedness: for example, given a human sequence, the yeast ortholog will be weighted more heavily in deriving the probability vector than the chimp sequence because the human and chimp sequences have had very little time to diverge compared to human and yeast, and their mutually conserved positions are due more to recent common ancestry than to negative selection. It also allows a more general definition of “conservation pattern,” as a profile can represent conservation of a single amino acid (e.g., high probability for only methionine) or conservation of a class of amino acids (e.g., high probabilities for only hydrophobic amino acids). To score the substitution of amino acid *b* by amino acid *a,* the subPSEC score uses the position-specific probabilities of *a* and *b* in the profile, according to the equation: subPSEC = ln(P_a_/P_b_), so that as P_a_ becomes smaller compared to P_b_, subPSEC becomes increasingly negative. Smaller subPSEC scores therefore predict a higher probability of a deleterious functional effect.

The critical assumption in the subPSEC method is that the evolutionary constraints are the same across the sequences used to build the amino acid profile. This is generally accepted to be approximately true for orthologous sequences (sequences related by a speciation event) and is the basis for complementation experiments. Whether orthologs or paralogs, more closely related sequences are more likely to have similar functional constraints on their evolution but, as described above, are less useful statistically because of their recent common descent. We have therefore modified the original subPSEC method to perform position-specific phylogenetic sampling [6]. In this method, the set of sequences used to build the amino acid profile can be different for each position. If the position is variable among orthologs, or conserved among orthologs but not paralogs, then only orthologs are used to calculate the profile because a sequence divergence from other subfamilies of paralogous proteins may be due to functional divergence. However, if the same amino acid is conserved in all the orthologous sequences as well as in the closest paralogous subfamily, we can add the paralogous sequences to the set used to calculate the amino acid profile. In this case, we can assume that the evolutionary constraints are similar, at least for that position. We can therefore use statistics from paralogous proteins when the same amino acid is conserved at the same position. This is done iteratively until a position is found either to be divergent in a subtree or to be conserved across all paralogs in the tree. We find that the diversity of sequences over which a position has been conserved (n_ic_, or number of independent counts) is a useful term to add to the subPSEC score, in addition to the probabilities of the amino acids P_a_ and P_b_. We performed log-linear logistic regression to obtain the best discrimination between human disease-causing variation and normal human variation (HGMD versus dbSNP, as described [7]), obtaining estimates and standard deviations for the coefficients in the equation:


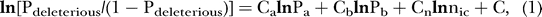


where *a* is the less probable amino acid and *b* is the more probable. The coefficients and standard deviations in their estimates were C_a_ = 0.89 ± 0.03, C_b_ = −0.88 ± 0.03, C_n_ = 0.94 ± 0.04, C = 3.00 ± 0.13. For this study, subPSEC was calculated as: 


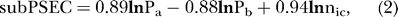


meaning that a cutoff of −3 corresponds to a 50% probability that the score came from HGMD (presumably mostly deleterious) or dbSNP (presumably mostly neutral). This allows us not only to calculate a subPSEC score but also to convert that score into a probability of deleterious functional effect P_deleterious_ from Equation 1.

The distribution of random subPSEC scores for ABCA1 were calculated as described previously [7]. Briefly, all single nucleotide substitutions in the coding sequence of *ABCA1* were generated, and those that led to amino acid substitutions were assigned subPSEC scores. The distribution was obtained by weighting each amino acid substitution according to the transition/transversion probabilities of the corresponding nucleotide change.

### Generation of stable cell lines.

Polyclonal stable cell lines expressing *ABCA1* sequence variants were generated using the Flp-in system (Invitrogen, Carlsbad, California, United States) as previously described [23]. The generation and detailed biochemical characterization of many of these cell lines are described elsewhere (R. Singaraja, H. Visscher, E. R. James, G. Chimini, and M. R. Hayden, unpublished data). Briefly, the nucleotide mutations were incorporated into a human *ABCA1* cDNA using PCR-based site-directed mutatgenesis as previously described [24] (primer sequences and PCR protocols are available on request) and cloned into the pcDNA5/FRT expression vector (Invitrogen). All plasmids were completely sequenced prior to transfection. Stable cell lines were generated by cotransfecting human embryonic kidney 293 Flp-in cells (Invitrogen) with the mutation-harboring plasmid and the pOG44 plasmid (Invitrogen). Transfected cells were maintained in DMEM (GIBCO, San Diego, California, United States) supplemented with 10% FCS, l-glutamine, and penicillin and streptomycin. Hygromycin-resistant colonies were selected for in 75 μg/ml Hygromyocin (Invitrogen), trypsinized, and pooled to generate polyclonal cell lines.

### Western blotting and RT-PCR.

ABCA1 expression was determined by Western blotting, as previously described [25]. Briefly, cells were lysed in 20 mm HEPES, 5 mm KCl, 5 mm MgCl_2_, 0.5% (v/v) Triton X-100, and complete protease inhibitor (Roche, Basel, Switzerland), and protein concentration was determined by the Lowry assay. Equivalent amounts of total protein were separated by SDS-PAGE, transferred to PVDF membranes, and probed with anti-ABCA1 [25] or anti-GAPDH (Chemicon, Temecula, California, United States) antibodies.

RNA was isolated from cells using TriZOL reagent (Life Technologies, Carlsbad, California, United States), and 3 μg of total RNA was reverse-transcribed using Superscript II (Life Technologies). RT-PCR was performed using previously described primers and protocols [26].

### Cholesterol efflux.

Efflux experiments were performed as previously described [23]. Briefly, cells were loaded overnight with 1 μCi of [^3^H]cholesterol (Amersham Biosciences, Little Chalfont, United Kingdom) in DMEM supplemented with 10% FCS, l-glutamine, and penicillin and streptomycin. The following day, the medium was removed and replaced with serum-free medium containing 0.2% delipidated bovine serum albumin (Sigma, St. Louis, Missouri, United States). After a 1-h incubation, 10 μg/ml human apoA-I (Athens Research and Technology, Athens, Georgia, United States) was added. After 4 h, the medium was removed and centrifuged, and cells were lysed in 0.2% SDS. The amount of [^3^H]cholesterol in the supernatant and cells was determined by liquid scintillation spectroscopy. Cholesterol efflux values are the mean of at least three separate assays, each performed in triplicate, and are presented as mean ± SD of the difference between efflux in the presence and absence of apoA-I. Each assay was performed together with wild-type ABCA1, and values are expressed as percent of wild-type efflux. Significance was calculated using a one-way ANOVA test with a Newman-Keuls post-test using GraphPad Prism 4 software (San Diego, California, United States).

## Supporting Information

Figure S1 Expression of the S1731C Allele in Polyclonal Stable Cell Lines ABCA1 protein (A) and mRNA (B) expression levels were determined in an untransfected control cell line, and cells transfected with wild-type ABCA1 or the S1731C variant. The cell line transfected with the S1731C allele expressed low levels of protein (A), but normal levels of mRNA (B), indicating that this variant impairs ABCA1 function by inhibiting the generation of a stable protein.
(37 KB PDF)

### Accession Numbers

The Online Mendelian Inheritance in Man (http://www.ncbi.nlm.nih.gov/OMIM) accession number for TD is 2054000. The PANTHER HMM accession numbers (http://www.pantherdb.org) are ABCA transporter family (PTHR19229) and ABCA1 subfamily (PTHR19229:SF34). The Entrez accession numbers (http://www.ncbi.nlm.nih.gov/entrez) are *ABCA1* (19), *ABCA7* (10347), *ABCA4* (24), and *ABCA2* (20).

## Abbreviations

*ABCA1*: ATP-binding cassette transporter A1
apoA-I: apolipoprotein A-I
cSNP: coding single nucleotide polymorphism
HDL: high density lipoprotein
HMM: hidden Markov model
subPSEC: substitution position-specific evolutionary conservation
TD: Tangier disease
