# Supplementary material for: Accurate Prediction of the Functional Significance of Single Nucleotide Polymorphisms and Mutations in the ABCA1 Gene
Source: PLoS Genet. 2005 Dec 30;1(6):e83. doi: 10.1371/journal.pgen.0010083 (PMC1342637; doi:10.1371/journal.pgen.0010083)
Supplement: Figure S1 — ABCA1 protein (A) and mRNA (B) expression levels were determined in an untransfected control cell line, and cells transfected with wild-type ABCA1 or the S1731C variant. The cell line transfected with the S1731C allele expressed low levels of protein (A), but normal levels of mRNA (B), indicating that this variant impairs ABCA1 function by inhibiting the generation of a stable protein. (37 KB PDF) [file pgen.0010083.sg001.pdf]

A

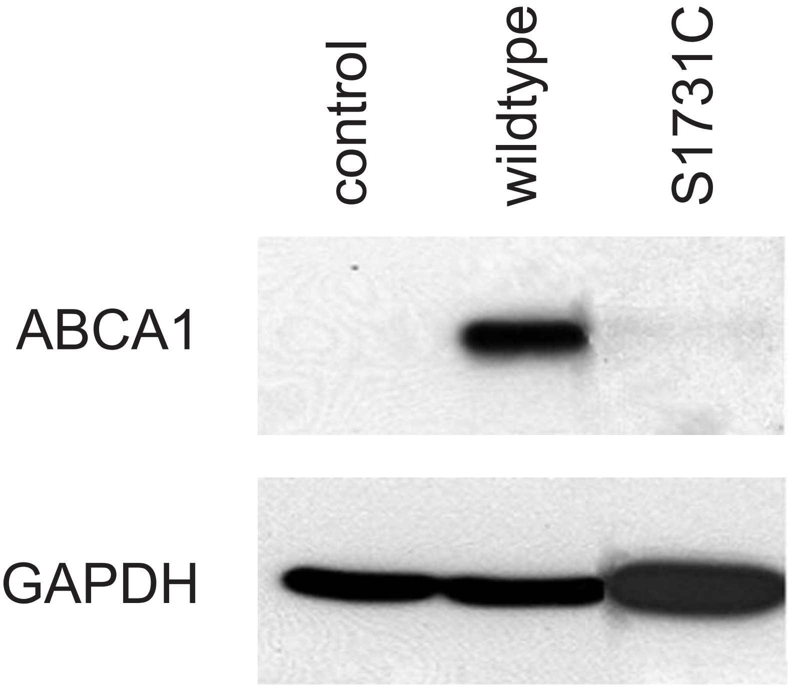

B

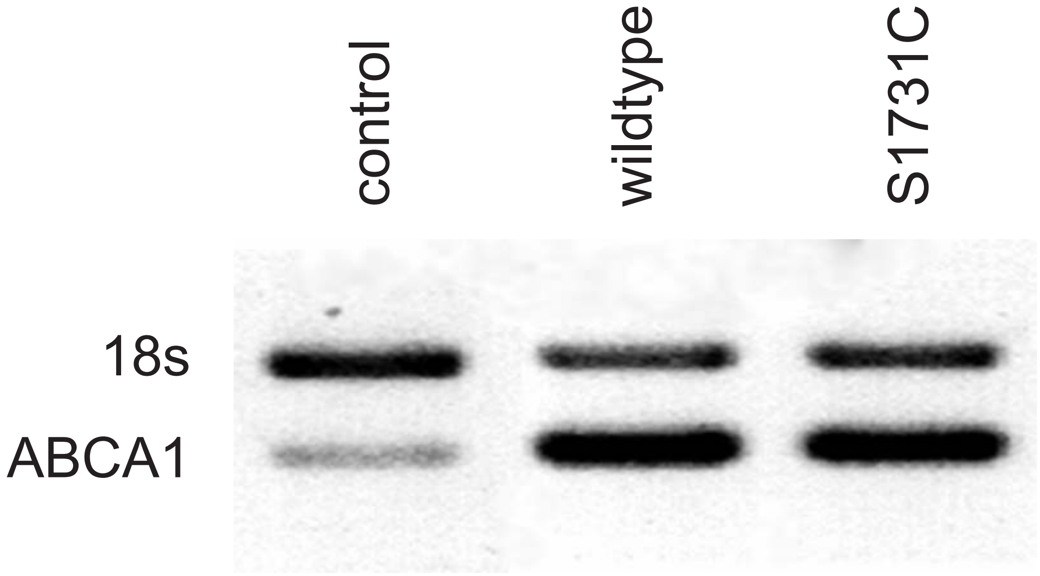

Supplemtal Figure 1

Supplemental Figure 1. Expression of the S1731C allele in polyclonal stable cell lines. ABCA1 protein (A) and mRNA (B) expression levels were determined in an untransfected control cell line, and cells transfected with wildtype ABCA1, or the S1731C variant. The cell line transfected with the S1731C allele expressed low levels of protein (A), but normal levels of mRNA (B), indicating that this variant impairs ABCA1 function by inhibiting the generation of a stable protein.
